# Vitamin D Receptor Polymorphism among Rickets Children in Mongolia

**DOI:** 10.2188/jea.17.25

**Published:** 2006-12-29

**Authors:** Akie Kaneko, Vaanchig Urnaa, Keiko Nakamura, Masashi Kizuki, Kaoruko Seino, Tomoko Inose, Takehito Takano

**Affiliations:** 1 Health Promotion, Division of Public Health, Graduate School of Tokyo Medical and Dental University.; 2 School of Public Health, Mongolian Health Science University.; 3 International Health and Medicine, Division of Public Health, Graduate School of Tokyo Medical and Dental University.

**Keywords:** Vitamin D, Receptors, Calcitriol, Polymorphism (Genetics), Rickets, Mongolia, tibial cortical speed of sound

## Abstract

**BACKGROUND:**

It was reported that 32% of children under five years old in Mongolia had symptoms of rickets. Vitamin D receptor (VDR) gene polymorphism has received attention in relation to bone metabolism. We therefore investigated whether VDR polymorphism is related to high prevalence of rickets in Mongolia and to bone properties in childhood.

**METHODS:**

We conducted a case-control study in Ulaanbaatar involving 80 children aged 7-10 years with a history of rickets (cases) and 72 children with no history of rickets (controls). VDR polymorphism was assessed using *Bsm*I*, Apa*I*,* and *Taq*I*,* and bone properties were determined by measuring age-standardized midtibial cortical speed of sound (TCSOS).

**FINDINGS:**

Each allelic frequency was verified to satisfy the Hardy-Weinberg equilibrium in cases, controls, and the total sample. The VDR polymorphisms among cases (BB 3%, Bb 18%, bb 80%; AA 15%, Aa 38%, aa 47%; and TT 81 %, Tt 17%, tt 3%) did not differ significantly from those among controls (BB 1%, Bb 13%, bb 86%; AA 16%, Aa 46%, aa 38%; and TT 86%, Tt 13%, tt 1%). There were no significant differences in TCSOS according to the VDR genotype among either cases or controls.

**CONCLUSIONS:**

The VDR polymorphism does not play a major role in the development of rickets in Mongolia and has no effect on TCSOS in childhood.

Prevalence of rickets in Mongolia in 2000 was reported to be 32% among children under five years old according to UNICEF, the Mongolian Ministry of Health, and the Nutrition Research Center of Mongolia.^1^ These children had one or more of the clinical signs of rickets. Although this figure is lower than that reported by an earlier study, in which 45% of young children showed skeletal abnormalities typical of rickets,^2^ rickets is still a common childhood disease in Mongolia.

Several factors have been suggested as reasons to explain this high prevalence of rickets in Mongolia; limited sunlight exposure due to high latitude and cold weather, low nutritional situation, and genetic factors.^3^^-^^5^ With regard to genetic factors, vitamin D receptor (VDR) polymorphism is considered to have an association with rickets, although few studies have examined the association.

Point mutations in the VDR gene are identified in hereditary 1, 25-dihydroxyvitamin D3-resistant rickets, a rare but severe form of rickets.^6^ VDR gene polymorphism, the milder nucleotide variations that do not cause specific disease, has received attention in relation to the risk of osteoporosis and to bone mineral density since first reported by Morrison et al.^7^ Given the possible associations between bone metabolism and the VDR polymorphism, rickets may be related to the VDR gene polymorphism.

The contributions of genetic factors to rickets in Mongolia have not been examined despite such a high prevalence of the disease. We therefore investigated the VDR polymorphism among Mongolian children and examined the hypothesis that the VDR polymorphism is associated with high prevalence of rickets in Mongolia and with bone properties in childhood.

## METHODS

### Subjects

The present study was performed in 80 children aged 7-10 years old with a history of rickets (cases) and in 72 children with no history of rickets (controls). To have an 80% chance of detecting a significant difference (p=0.05, two-sided), on the assumption that the frequencies of genotypes BB and Bb would be 30% among cases and 10% among controls, a minimum of 62 children were required for each study group. We enrolled additional children in each group to compensate for some non-evaluable subjects. Both cases and controls were selected from the children's roster with consultation records filed at a health center in Ulaanbaatar, Mongolia. None of the subjects were diagnosed as having hereditary 1, 25-dihydroxyvitamin D3-resistant rickets or disease-associated rickets.

The health center that participated in the present study was one of the 9 district health centers of the city of Ulaanbaatar and covered a population of 169,000. The center was a public facility with physicians practicing family medicine. Both preventive and primary curative services, including maternal and child health services, were provided for people living in the area. The children's roster used in this study consisted of children who visited the health center not only for the treatment of diseases but also for health consultations.

Children with a history of rickets and those with no history of rickets were invited to participate in a survey examination according to consecutive order on the roster. Children and their primary caretakers, mainly their mothers, joined the survey examination during the period from February through March 2003. Written informed consent was obtained from the principal caretakers of all subjects, and oral consent was obtained from all children participating in the present study. The ethical appropriateness of the survey protocol and DNA analysis was approved by the Mongolian National Medical University.

### Diagnosis of Rickets

The presence of at least one of the following clinical signs was used for diagnosis of rickets in Mongolia: craniotabes, rachitic rosary, Harrison's groove, delayed closure of fontanelle, muscular hypotonia, abdomen of a frog, spinal deformation, pigeon chest, or bowed legs. Family doctors at the health center recorded a diagnosis of rickets according to these criteria and these records were used for selection of cases and controls.

### VDR Genotypes

Peripheral venous blood was collected in EDTA tubes at the health center, frozen at －20 °C, and transferred to Tokyo Medical and Dental University. DNA of peripheral white blood cells was amplified by polymerase chain reaction (PCR) and examined using the restriction fragment length polymorphism (RFLP) method. VDR genotypes of each subject were identified according to the digestion pattern and alleles according to the presence (b, a, and t) or absence (B, A, and T) of the *Bsm* I*, Apa*I*,* and *Taq*I sites.

### Bone Properties

Subjects' bone properties were assessed during the survey period by the quantitative ultrasonic measurement (QUS). Speed of sound of tibial cortical bone (TCSOS) was measured using a SoundScan Compact 2000™ (Omron Myriad Ultrasound Systems Ltd., Tokyo, Japan), along the cortex of the right tibia at the midtibial point. The age-standardized value of TCSOS (standardized TCSOS) was calculated for analysis of bone properties by applying Asian standards for boys and girls.^8^

### Statistical Analysis

Chi-square test was used to examine whether allelic distribution of RFLPs satisfied the Hardy-Weinberg equilibrium. Distribution of VDR genotypes was compared using chi-square test, and standardized TCSOS was analyzed by either one-way analysis of variance (ANOVA) or t-test. A p-value of less than 0.05 was considered to indicate statistical significance.

## RESULTS

The VDR genotypes were identified from the blood samples of 79 cases and 69 controls. Each allelic frequency was verified to satisfy the Hardy-Weinberg equilibrium in cases, controls, and the total sample. The distribution of each allele was as follows: B (0.11, 0.08), b (0.89, 0.92), A (0.34, 0.39), a (0.66, 0.61), T (0.89, 0.92), and t (0.11, 0.08) (allelic frequency in cases, in controls).

The distribution of VDR genotypes among children in Ulaanbaatar is shown in Table 1. The most common genotypes were bb, aa, and TT in cases, and bb, Aa, and TT in controls. When the genotypes for all polymorphisms were combined, the most frequent genotypes were bbaaTT in cases, and bbaaTT and bbAaTT in controls. Chi-square tests detected no significant differences in the distribution of VDR genotypes among cases and controls.

**Table 1.** Distribution of vitamin D receptor (VDR) genotypes among children with a history of rickets (cases) and those with no history of rickets (controls) in Ulaanbaatar.

VDR genotype		Cases (%)	Controls (%)	Total (%)	P value^*^
Total		79 (100)	69 (100)	148 (100)	

*Bsm*I	bb	63 (80)	59 (86)	122 (82)	0.644
	Bb	14 (18)	9 (13)	23 (16)	
	BB	2 (3)	1 (1)	3 (2)	

*Apa*I	aa	37 (47)	26 (38)	63 (43)	0.507
	Aa	30 (38)	32 (46)	62 (42)	
	AA	12 (15)	11 (16)	23 (16)	

*Taq*I	tt	2 (3)	1 (1)	3 (2)	0. 744
	Tt	13 (17)	9 (13)	22 (15)	
	TT	64 (81)	59 (86)	123 (83)	

Homozygous	bbaaTT	36 (46)	26 (38)	62 (42)	0.712
	bbAATT	5 (6)	7 (10)	12 (8)	
	BBAAtt	2 (3)	1 (1)	3 (2)	
Heterozygous	bbAaTT	22 (28)	26 (38)	48 (32)	
	BbaaTT	1 (1)	0 (0)	1 (1)	
	BbAaTt	8 (10)	6 (9)	14 (10)	
	BbAATt	5 (6)	3 (4)	8 (5)	

* : Results of chi-square tests to examine the distribution of VDR genotypes among children with and without a history of rickets

Table 2 shows the standardized TCSOS in both groups (79 cases and 68 controls) compared according to the VDR genotype. No significant differences in standardized TCSOS were found according to the VDR genotype in either group. Standardized TCSOS among cases ( －0.536 + 0.136, mean ± standard error) was significantly lower than that among controls ( －0.037 + 0.140) (p=0.012).

**Table 2.** Standardized speed of sound of midtibial cortical bone (TCSOS) of prepubertal schoolchildren in Ulaanbaatar by vitamin D receptor (VDR) genotype.

		Standardized TCSOS^*^							

		Cases (n=79)				Controls (n=68)			
	
VDR genotype		n	Mean	SEM^**^	P value**^†^**	n	Mean	SEM^**^	P value**^†^**
*Bsm*I	bb	63	-0.595	0.158	0.692	58	-0.025	0.157	0.875
	Bb	14	-0.320	0.283		9	-0.168	0.328	
	BB	2	-0.201	0.349		1	0.413	-	

*Apa*I	aa	37	-0.621	0.224	0.841	26	-0.122	0.240	0.833
	Aa	30	-0.473	0.196		31	0.056	0.218	
	AA	12	-0.431	0.318		11	-0.102	0.256	

*Taq*I	tt	2	-0.201	0.349	0.696	1	0.413	-	0.875
	Tt	13	-0.312	0.306		9	-0.168	0.328	
	TT	64	-0.592	0.156		58	-0.025	0.157	

Total		79	-0.536	0.136		68	-0.037	0.140	

* : Standardized by TCSOS dataset of an Asian standard population

**: Standard error of the mean

† : Results of t-tests to examine the differences in standardized TCSOS by VDR genotype

## DISCUSSION

The present study is the first to report the distribution of VDR polymorphisms and TCSOS among children in Ulaanbaatar, Mongolia. The frequencies of the VDR genotypes did not differ significantly according to a history of rickets, and VDR polymorphism also showed no association with TCSOS among children with or without a history of rickets.

The subjects in this study were selected from a list of children who had health consultation records including a diagnosis of rickets. The list was reported to have covered 90% of the children in the district.^9^ The reliability and validity of TCSOS as a measurement of bone properties have been confirmed previously not only among healthy subjects^10^^-^^12^ but also among subjects with bone disorders.^13^^,^^14^ The TCSOS was thus considered useful for the evaluation of bone properties of the subjects in this study.

Our results did not indicate significant differences in VDR genotype distribution among children with and without a history of rickets. Accordingly, we analyzed the distribution of VDR genotypes among children in Ulaanbaatar collectively and found very low frequencies of the alleles B (0.098) and t (0.095), which are thought to be disadvantageous for bone turnover.^15^ With regard to the *Bsm*I genotype, distribution among this population indicated the predominant frequency of bb (82%), with very low frequency of BB (2%). This distribution is similar to Japanese (bb: 77%, BB: 1%),^16^ Korean (bb: 86%, BB: 1%),^17^ and Chinese (bb: 90%, BB: 1%) populations,^18^ but different from Caucasian populations (bb: 28-53%, BB: 17-26%).^19^^-^^22^

The results of the present study did not support associations between the VDR genotype and the TCSOS in prepubertal schoolchildren in Ulaanbaatar, Mongolia. It is still controversial whether the VDR polymorphism is associated with bone properties. Some studies showed that the B allele of the *Bsm*I-RFLP site was significantly associated with lower BMD,^19^ while others failed to find such an association,^20^^,^^21^ or found contradictory results, where the b allele was associated with low BMD.^22^ However, these conflicting results do not mean that there is no relation between VDR polymorphism and bone properties. A meta-analysis suggested that the inconsistencies in the results regarding the association between VDR genotype and bone properties are the results of various influences of non-genetic factors on bone properties.^15^ Further studies that consider the influences of non-genetic factors together with those of VDR polymorphism on bone properties would broaden our understanding of the role of VDR polymorphism in bone metabolism.

Lower mineral density among rickets children than healthy children was previously reported.^23^ Our study revealed that prepubertal schoolchildren in Ulaanbaatar with a history of rickets showed a significantly lower TCSOS than those with no history of rickets. As the TCSOS reflects strength and elasticity of bone,^24^ our results indicated weaker bone properties of rickets children than others. Weak bone properties in prepubertal school-age are disadvantageous in gaining a high level of peak bone mass resulting in reduced bone development later in life. Thus, high prevalence of rickets is a critical concern among Mongolian children.

In conclusion, the VDR polymorphism itself does not play a major role in high prevalence of rickets in Mongolia, nor in reduced speed of sound of tibial cortical bone in childhood. In developing preventive measures of rickets in Mongolia, non-genetic causes of the disease should be further studied.
